# Evaluation of the utility of teaching joint relocations using cadaveric specimens

**DOI:** 10.1186/s12909-018-1151-0

**Published:** 2018-03-20

**Authors:** John Au, Edward Palmer, Ian Johnson, Mellick Chehade

**Affiliations:** 1 The University of Adelaide, Frome Road, Adelaide, South Australia SA 5005 Australia; 2 Discipline of Anatomy and Pathology, Adelaide Medical School, The University of Adelaide, Frome Road, Adelaide, South Australia SA 5005 Australia; 3 Royal Adelaide Hospital, The University of Adelaide Centre for Orthopaedic Trauma and Research, North Terrace Adelaide, Adelaide, South Australia 5000 Australia

**Keywords:** Cadaver, Medical student, Education, Online learning, Joint dislocation, Joint relocation

## Abstract

**Background:**

Like other procedural skills, the ability to relocate a joint is an important aspect of junior doctor education. Changes in the approach to teaching and learning from the traditional apprenticeship-style model have made the teaching of practical skills more difficult logistically. Workshops utilising cadaveric specimens offer a solution to this problem.

**Methods:**

One hundred forty-six fourth year medical students were randomly divided into 5 groups. Each group received a different teaching intervention based on ankle, patella and hip relocation. The interventions consisted of online learning modules, instructional cards and workshops using skeleton models and cadaveric dislocation models. Following the intervention students were given a test containing multiple choice and true/false style questions. A 13-item 5-point Likert scale questionnaire was also delivered before and after the intervention. The data was analysed using one-way analysis of variance (ANOVA) and the Bonferroni post-hoc test.

**Results:**

Compared to the instructional cards group, the other 4 groups showed a 10.8–19.2% improvement in total test score (*p* < 0.01) and an 18.4–25.3% improvement in self-reported understanding and confidence in performing joint relocations (*P* < 0.01). There was no significant difference in total test scores between groups exposed to cadaveric instruction on the relocation of one-, two- or all three- joints, nor any significant difference between all the cadaveric dislocation groups and the group receiving instruction on the skeleton model.

**Conclusion:**

The results of the present study suggest that workshops utilising cadaveric dislocation models are effective in teaching joint relocation. In addition, the finding that lower fidelity models may be of equal utility may provide institutions with flexibility of delivery needed to meet financial and resource constraints.

## Background

Joint dislocations are a common reason for orthopaedic referral with a lifetime risk of one in sixteen for traumatic dislocations [1]. Other studies report an annual incidence of 42.1 cases per 100,000 persons [2]. The management of dislocation by joint relocation usually involves the application of joint traction to overcome muscle spasms and regain muscle length [3].

With most dislocations, prompt relocation of the joint is crucial. If a shoulder dislocation is not assessed and treated immediately, more time is allowed for muscle spasms to occur making it more difficult to relocate [4]. In a hip dislocation, the bone can directly compress the sciatic nerve resulting in acute nerve ischaemia with irreversible nerve injury if not reduced emergently [5]. In addition there is an increased risk of avascular necrosis of the femoral head due to the compromised blood supply in the dislocated position. In our institution many cases of dislocations (particularly ankle) are transferred from rural hospital centres (often many hours away) still dislocated with threatened medial skin and articular surface viability because of a lack of training and unwillingness of medical officers to attempt relocation (local trauma audit – Royal Adelaide Hospital).

According to the Australian Curriculum Framework for Junior Doctors and Australian Musculoskeletal Education Collaboration, joint relocation is a procedure that junior doctors should be able to competently perform [6, 7]. In the US, emergency medicine residents are expected to perform at least 10 reductions as determined by the Accreditation Council for Graduate Medical Education [8]. Yet, a survey of UK junior doctors found they lacked experience with this procedure and were unable to perform a joint relocation without support. [9]. Indeed, there has been a decline in experience across a wide range of procedural skills [10, 11]. A recent survey of 664 graduating medical students in the US revealed that 33% had never intubated a patient, 30% had never inserted a nasogastric tube, 28% had never drawn blood gases and 5% had never sutured [12]. These findings are consistent with what is being reported by graduating medical students in Australia and this has not changed in the past two decades [13, 14].

There is a traditional assumption that students learn procedural skills during clinical placements and formal teaching in the curriculum is not required [15]. As a result, students mostly obtain their procedural skills on the wards from residents and registrars in an opportunistic, ad hoc manner [14, 16]. This style of teaching may have worked in the past, but changes to healthcare systems across the world have made it less feasible. Technological advancements in many areas of medicine result in patients spending less time in hospital. Pressure to increase productivity and treat patients within a certain timeframe force senior doctors to perform more procedures themselves rather than teach and supervise [9]. Furthermore, a dramatic increase in medical student numbers has reduced the number of hospital patients available to each student [17]. These challenges and other reasons have resulted in students receiving fewer opportunities for hands-on practice and this may account for the current lack of experience with procedural skills seen in graduating doctors.

One solution to this problem is to develop procedural skills workshops utilising cadaveric specimens. This provides a safe and effective learning environment for students to practise basic procedural skills without involving real patients [17]. The cadavers offer in-situ anatomy, realistic tissue handling and haptic feedback making it an excellent training model [18]. Studies have confirmed the efficacy of such workshops with students reporting an increase in confidence and understanding of the procedures taught [10, 11, 19].

The aim of this study was to assess the utility of teaching joint relocation using cadaveric models and to compare it to other methods commonly used, such as online learning modules and visual information cards containing written instructions. In particular, we examined whether providing students with dislocation models that allow for hands-on practice would better improve their understanding and confidence with joint relocations. To our knowledge this is the first study to create cadaveric models that allow students to attempt relocating a dislocated joint.

## Methods

### Online module development

Three online modules were created covering ankle, patella and hip dislocation. Each module contained relevant anatomy sourced from the textbook Clinically Oriented Anatomy [20]. Relocation techniques covered in the modules were derived from Orthopaedic Emergencies: Expert Management for the Emergency Physician [21].

### Relocation instruction card

For each joint dislocation the steps for relocation along with an instructional photograph were printed onto an A4 piece of paper and laminated. The photograph demonstrated correct patient positioning, correct performer positioning and correct hand positioning. It also included arrows to indicate the correct direction to apply traction and counter traction.

### Cadaveric model development

#### Ankle dislocation model

**Fig. 1:**
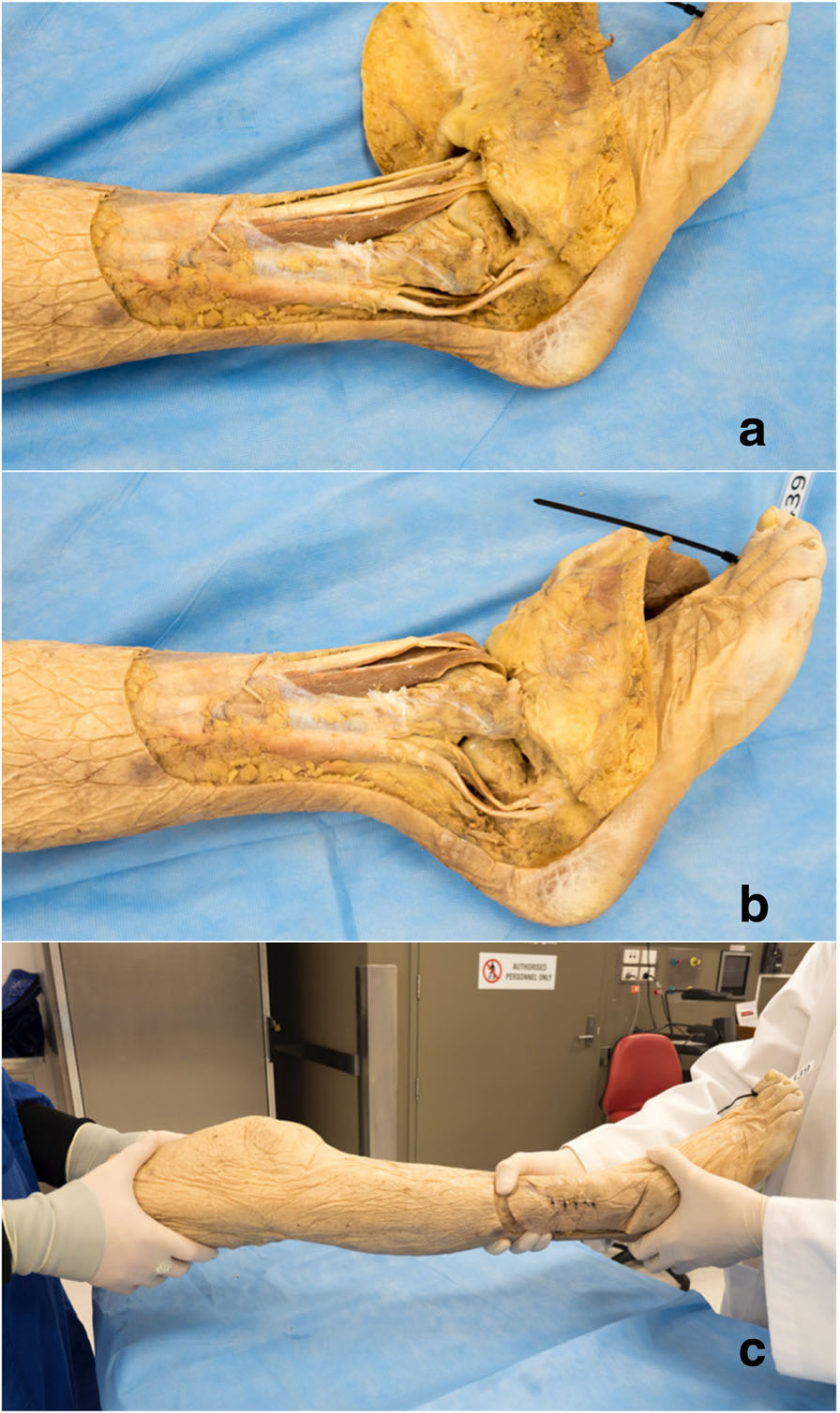
**a** through **c**: Ankle Dislocation Model. Lateral views of the ankle with lateral ligaments cut (**a**) and ankle dislocated (**b**). Ankle relocation procedure is demonstrated in (**c**)

An incision was made around the medial and lateral malleoli joining at the mid shin region and the skin reflected back (Fig. 1). Subcutaneous tissue surrounding the ankle joint was removed to expose the ankle joint. The anterior and posterior parts of the joint capsule were then divided. The lateral ankle ligaments (anterior talofibular, calcaneofibular and posterior talofibular) and medial ankle ligaments (anterior and posterior tibiotalar, tibiocalcaneal, tibionavicular) were also divided.

#### Patella dislocation model

**Fig. 2:**
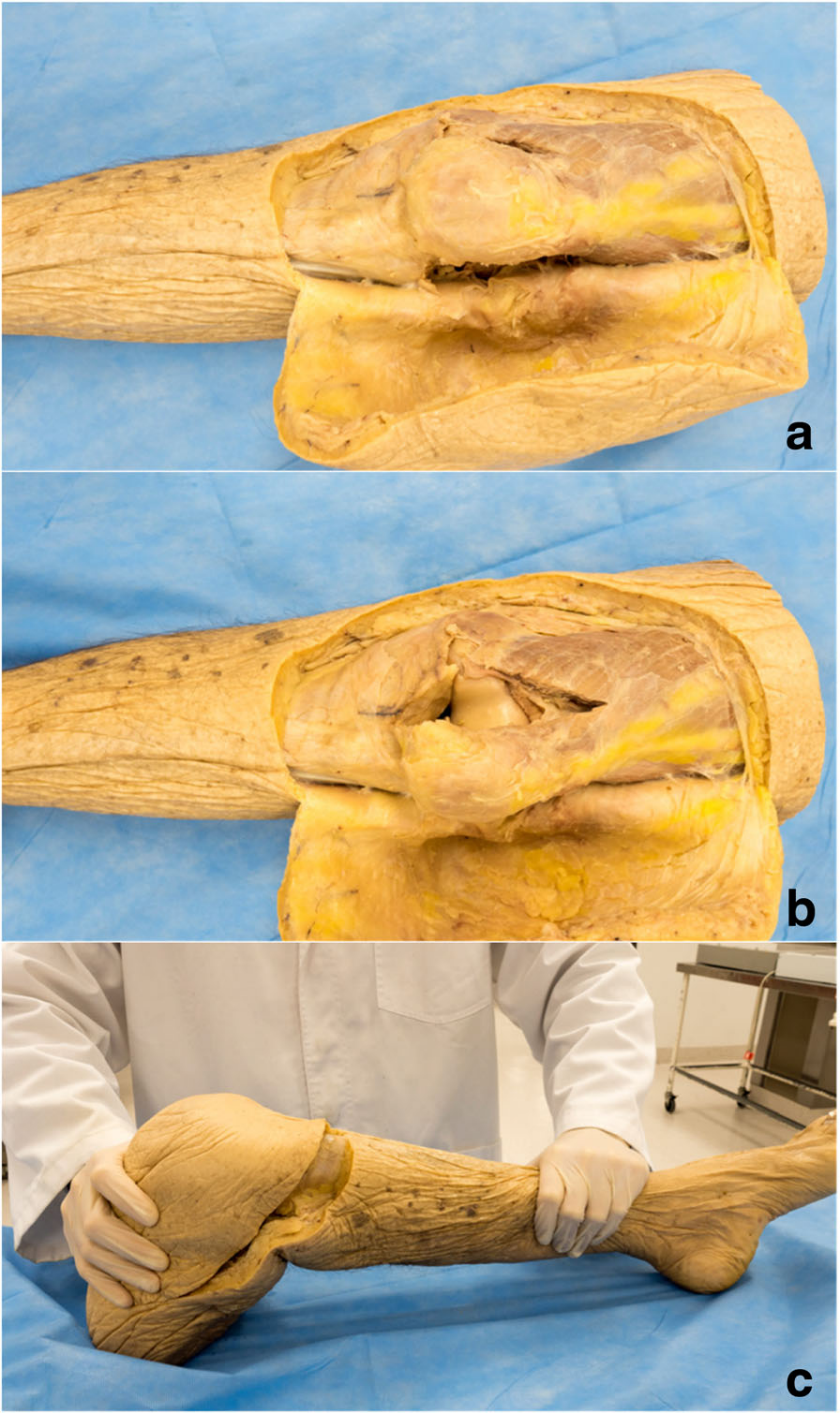
**a** through **c**: Patella Dislocation Model. Anterior views of the knee with vastus medialis obliquus cut (**a**) and patella dislocated (**b**). Patella relocation procedure is demonstrated in (**c**)

An incision was made above and below the knee joint meeting at the lateral knee and the skin was reflected back (Fig. 2). Subcutaneous tissue around the knee was removed to expose the knee joint and vastus muscles. The vastus medialis obliquus was divided at the muscle tendon junction to separate its attachment to the patella. The medial retinaculum and patellofemoral ligament were also divided to allow for dislocation.

#### Hip dislocation model

**Fig. 3:**
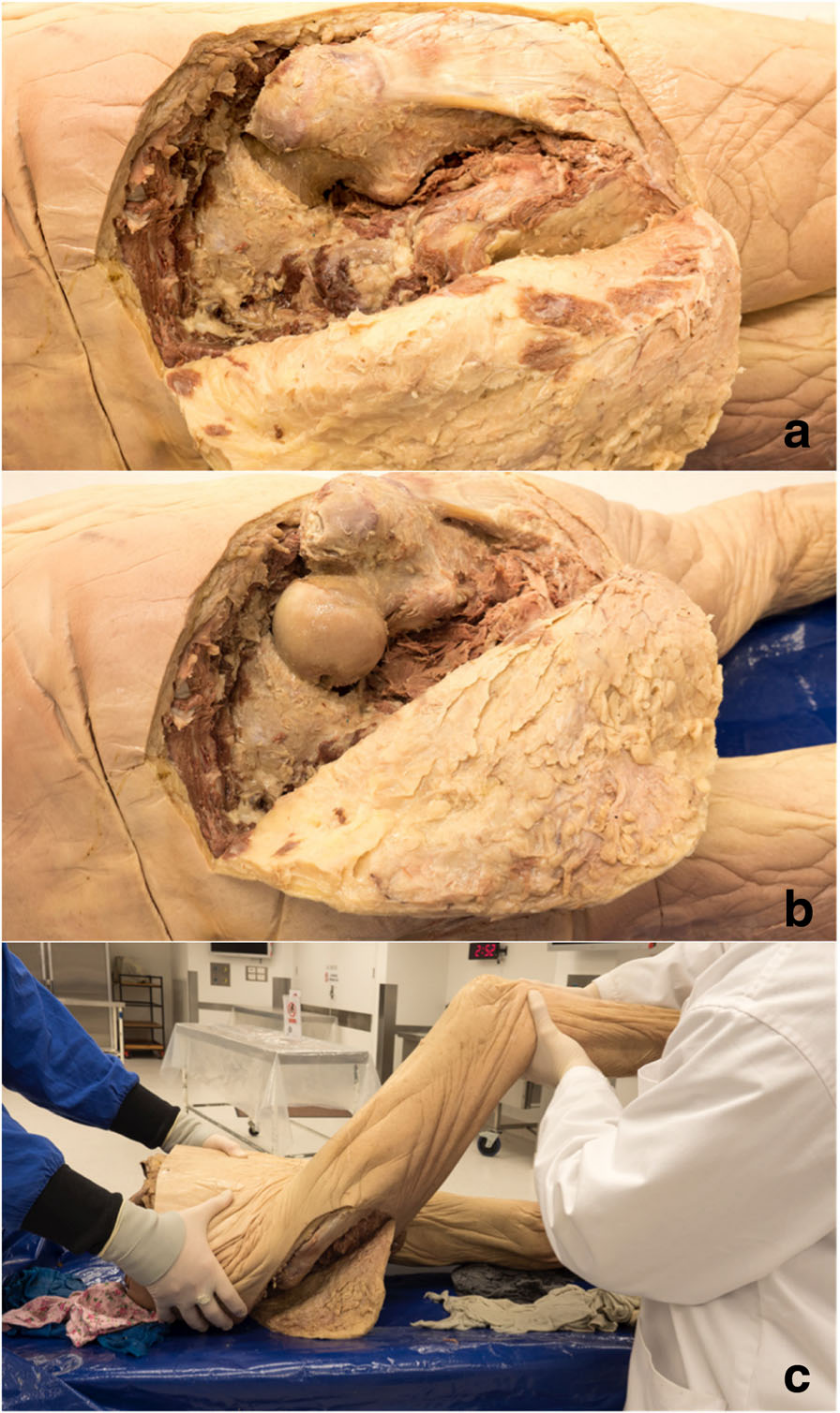
**a** through **c**: Hip Dislocation Model. Posterior views of the hip with gluteal muscles and hip ligaments removed (**a**) and hip dislocated (**b**). Hip relocation procedure is demonstrated in (**c**)

The cadaver was placed in prone position. An incision was made around the buttock and upper thigh and the skin was reflected back (Fig. 3). Superficial fascia from the fascia lata in the gluteal region was removed. Muscles of the gluteal region (piriformis, superior gemellus, inferior gemellus, quadratus femoris, gluteal medius and gluteal minimus) were removed to allow access to the hip joint. The ischiofemoral, iliofemoral, pubofemoral ligaments and the ligament of the head of femur were divided to disarticulate the hip.

#### Study design

**Fig. 4:**
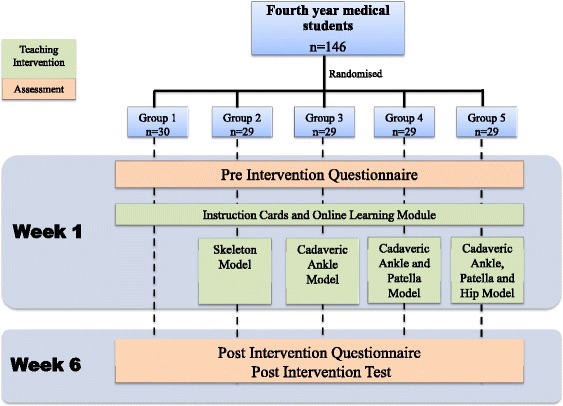
Study design flow chart

As part of the Bachelor of Medicine and Bachelor of Surgery (MBBS) program at The University of Adelaide, fourth year medical students (*n* = 146) were allocated into 5 groups based on consecutive rotations through the Musculoskeletal Medicine Program (Table 1). Students were randomly assigned to these groups by a Clinical Placements Team at the start of the year and each group experienced a 6 week Musculoskeletal Medicine Rotation. During this rotation a teaching intervention was delivered in week 1. The teaching intervention consisted of an online learning module and a teaching session in the anatomy laboratory. All students had access to the online module and information cards to provide them with baseline understanding of relocations. However, during the teaching sessions each group was exposed to a different teaching resource and proceeded to self-directed learning (Fig. 4):

- The first group was provided only with instructional cards explaining how to perform the joint relocations. All subsequent groups received this teaching resource.
- The second group had access to a skeleton model. They were able to move the skeleton to practise relocation techniques and visualise how they might relocate the ankle, patella and hip joints.
- The third group had access to a dislocated ankle prosection. They were able to practice relocating the ankle joint using this cadaveric specimen.
- The fourth group had access to a dislocated patella prosection as well as the dislocated ankle prosection.
- The fifth group had access to a dislocated hip prosection as well as the dislocated knee and ankle prosections.

**Table 1:** Number of students in each group

Group	Intervention	No. of Students (*N* = 146)
1	Instructional cards and online modules only	30
2	Skeleton model	29
3	Cadaveric ankle model	29
4	Cadaveric ankle and patella models	29
5	Cadaveric ankle, patella and hip models	29

Assessment of the teaching intervention consisted of two parts:

1. A 13-item Pre- and Post- intervention questionnaire. This 5-point Likert scale intervention questionnaire was created by the authors; with options ranging from strongly disagree to strongly agree. Four main themes were assessed using this questionnaire: 1) perceived importance of joint relocation; 2) understanding of anatomy related to joint dislocation; 3) familiarity with joint relocation technique and 4) confidence in performing joint relocation. The questionnaire was delivered in weeks 1 and 6 of the students’ rotation.
2. A 30-item post-intervention test consisting of MCQ and True/False style questions dealing with each of the three joints studied (hip, patella and ankle). For each joint there were 5 multiple-choice questions and 5 true/false questions assessing relocation technique and relevant anatomy. This test was delivered in week 6.

The questionnaire and tests were delivered online via the University’s learning management system.

#### Statistical analysis

All data were de-identified, entered into an Excel spreadsheet and analysed using SPSS version 23. Two medical education experts and a senior orthopaedic surgeon reviewed the tests and questionnaire to ensure face and construct validity. The reliability of the tests and questionnaire was calculated using the alpha coefficient of internal consistency (Cronbach Alpha).

The scores for the 3 individual tests (hip, patella and ankle) were added together to produce a total test score out of 30. The individual tests and the total test scores for each group were analysed using one-way analysis of variance (ANOVA), followed by the Bonferroni test for post hoc analysis.

Latent variables were revealed by a factor analysis of the questionnaire results. Questions 1, 2, 3 and 4 formed one factor that assessed students’ perceived importance of joint relocation. The average results for questions 1, 2, 3 and 4 were added together to produce an ‘importance score’ for each questionnaire. Questions 5, 6, 7, 8, 9, 10, 11, 12 and 13 assessed the perceived understanding of anatomy related to joint dislocation, familiarity with joint relocation technique and confidence in performing joint relocation and together formed another dominant factor. The average results of these questions were added together to produce a ‘comfort score’ for each questionnaire. For each group, pre-intervention questionnaire importance and comfort scores were compared with post-intervention questionnaire importance and comfort scores.

## Results

A total of 144 (99%) students completed the 3 tests (ankle, hip and patella), 146 students (100%) completed the pre-intervention questionnaire and 137 (94%) completed the post-intervention questionnaire.

Factor analysis revealed a latent variable for questions 1–4 of the questionnaire with each item loading between 0.725 and 0.811 to that factor. Similarly questions 5–13 revealed a latent variable as each item loaded at 0.61 or better and 6 items above 0.8 to one factor.

The measuring instruments showed high reliability (Table 2). Values of 0.7–0.8 are regarded as satisfactory [22].

**Table 2:** Test and questionnaire reliability

Assessment Item	Cronbach alpha coefficient
Pre-Intervention Questionnaire Q1 – Q4	0.778
Pre-Intervention Questionnaire Q5 – Q13	0.924
Post-Intervention Questionnaire Q1 – Q4	0.671
Post-Intervention Questionnaire Q5 – Q13	0.892
Test	0.729

**Fig. 5:**
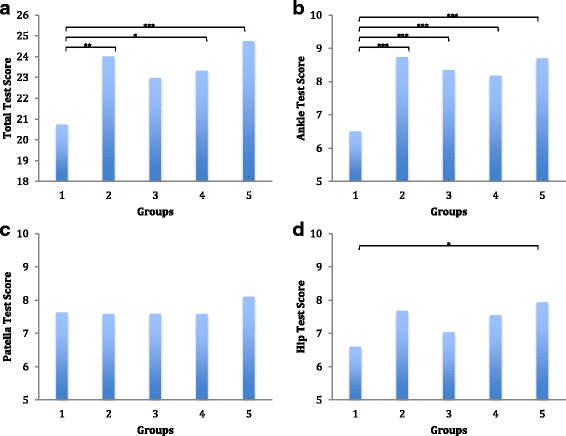
**a** through **d**: Column graphs of test scores (mean value) compared with group numbers. There was no significant difference in the patella test scores (*P* = 0.597) (**c**), but a significant difference was seen in total test scores (*P* < 0.001) (**a**), ankle test scores (*P* < 0.001) (**b**) and hip test scores (*P* = 0.009) (**d**)

One-way ANOVA showed a significant difference in total test scores between the groups (*P* < 0.001). Post hoc analysis showed that total test scores for groups 2, 4 and 5 were significantly higher than group 1 (*P* < 0.05) (Fig. 5); no significant difference was seen between groups 2, 3, 4 and 5. There was a significant difference in ankle test scores between the groups (P < 0.001). Post hoc analysis showed that ankle test scores for groups 2, 3, 4 and 5 were significantly higher than group 1 (P < 0.001); no significant difference was seen between groups 2, 3, 4 and 5. Analysis showed no significant difference in patella test scores between the groups (*P* = 0.597). Finally, there was a significant difference in hip test scores between the groups (*P* = 0.009). Post hoc analysis showed that hip test score for group 5 was significantly higher than group 1 (*P* = 0.012); no significant differences were seen between any of the other groups.

Aside from group 4, no significant increase in importance scores was seen between the pre- and post-intervention questionnaires (Table 3). However, all groups saw a significantly higher comfort score following the intervention (Table 4).

**Table 3:** Effect of intervention on importance score

Groups	Pre-intervention importance score	Post-intervention importance score	*P* Value for T test
1	17.87	17.13	0.276
2	17.10	18.07	0.229
3	17.86	18.31	0.425
4	16.76	18.12	0.015
5	17.61	18.19	0.276

**Table 4:** Effect of intervention on comfort score

Groups	Pre-intervention comfort score	Post-intervention comfort score	*P* Value for T test
1	22.53	30.27	0.001
2	15.07	35.83	0.001
3	18.24	37.15	0.001
4	18.38	36.68	0.001
5	19.79	37.93	0.001

**Fig. 6:**
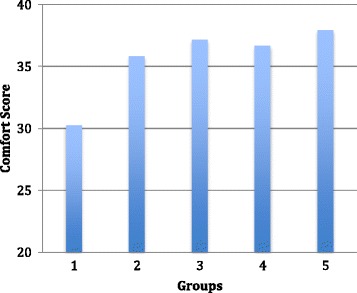
Column graph of comfort scores compared with group numbers. Comfort scores were significantly higher for groups 2, 3, 4 and 5 compared with group 1 (*P* < 0.001); no other significance found

One-way ANOVA showed a significant difference in post-intervention questionnaire comfort scores between the groups (*P* < 0.001). Post hoc analysis showed that groups 2, 3, 4 and 5 had a significantly higher post-intervention questionnaire comfort score compared to group 1 (P < 0.001); no other differences were seen between the groups (Fig. 6).

Overall, 97% of the students agreed or strongly agreed that the anatomy resource session with the cadaveric dislocation models was useful, while 86% agreed or strongly agreed that the online learning modules were useful.

## Discussion

The results of this study have demonstrated cadaveric specimens can increase understanding and confidence when used to teach joint relocation. We believe the results show that a purely theoretical approach to teaching this area without practical teaching sessions has inherent risks to both student understanding and confidence.

The total test scores for the groups utilising cadavers were higher than the control group whom only received pictures and written content. Group 3 who received the dislocated ankle prosection showed a 10.8% increase in total test score compared to the control group however this did not reach statistical significance. Group 4, who received a dislocated patella prosection as well as the dislocated ankle prosection, showed a 12.4% increase in total test score (*P* = 0.05). Group 5 who received all 3 dislocation prosections showed a 19.2% increase in total test score (*P* < 0.001). The cadavers allowed students to appreciate spatial orientation (where to position themselves) and handedness (which hand does what) when performing the procedure. They also provided a combination of visual and haptic feedback that allowed students to better memorise and recall motor patterns [23, 24].

An unexpected finding in the results was that even though there was a positive trend between groups 3, 4 and 5, no significant difference was found on post hoc analysis. An explanation for this is that even introducing a single joint dislocation model could increase student engagement in the learning process and thus increase their ability to retain information about the other joint dislocations as well. In addition, the basic principles of reducing muscle tension, providing traction and counter traction can be learnt with any joint relocation and may be readily transferable to other joints. Finally, the patella relocation technique consists of only 2 relatively simple steps making it easier to conceptualise. Consequently, a cadaveric patella dislocation model may not offer as great an advantage compared with less biofidelic methods of teaching patella relocation.

Another interesting finding is that the skeletal model performed just as well as the cadaveric models. Hamstra et al. offers an explanation by describing how low fidelity models can offer the same benefits as high fidelity models using constructivist theory [25]. Essentially learning a procedure involves objects and processes. Using shoe tying as an example, Hamstra et al. describes the shoe and lace as objects and the procedural knowledge of tying a knot as the process. By learning the process (knot tying) without regard to the object (shoes), the procedural knowledge or skill can be transferred (e.g to skates).

The importance score was derived from questionnaire questions 1–4, which included: ‘being able to relocate a joint is an important skill to have’ and ‘junior doctors should be able to perform joint relocations’. Students perceived joint relocation as very important in the pre-intervention questionnaires and this remained unchanged after the interventions were delivered. This suggests that students are entering their clinical years with a sound ability in recognising clinical relevance.

The comfort score was derived from questions such as: ‘I understand the anatomy involved with ankle relocation’, ‘I am familiar with the techniques involved with patella relocation’ and ‘I feel confident in regards to performing joint relocations on the hip’. There was a significant increase in comfort score following the intervention in each group. However, groups 2, 3, 4 and 5’s post-intervention comfort score was significantly greater than group 1. This indicates that having a skeletal model or cadaveric model to practise on significantly increases familiarity, understanding and confidence in regards to performing joint relocations.

### Limitations

A potential limitation in our methodology was that groups participated in this study and thus completed the test at different time points. This created the possibility for information sharing amongst students of different groups. Having all groups participate in the study at the same time would remove this limitation, however this was not feasible with the timetabling of the rotations. To reduce this variable, the students were specifically told that the test results had no effect on their rotation grading. When a test has low to moderate stakes the motivation to participate in information sharing is low [26].

Practical limitations involving the cadavers were also evident. Due to the embalming process the cadaveric tissue is much stiffer than tissue in an actual person. This made positioning the dislocation model and moving the joints more difficult. A solution to this problem could be to use different, softer, embalming methods. In addition, due to limitations on cadaver availability at our institution only ankle, patella and hip models were created.

We emphasise that junior doctors need to work within the limits of their competence. Whilst they may be expected to perform simple relocations such as shoulder and patella, more complex relocations such as ankle and hip would require further supervision and training. Nonetheless, junior doctors should only attempt such procedures under supervision and with the agreement of patients even if they feel confident following stimulated practice, as patient safety is paramount.

We also acknowledge that reported understanding and confidence may not necessarily translate into performance in future practice [27]. This is because there is a complex relationship between procedural confidence and competence. Confidence can act as a marker for competence but the correlation is poor [28, 29]. However, confidence is important because it independently affects performance [30]. It also influences a medical students willingness to undertake the procedure and ask for support [31]. In fact, lack of confidence is only second to lack of opportunity as the biggest barrier to performing procedures [32]. An interesting follow up study could try to determine if this cohort has any future exposure to dislocations in their clinical practice and their actions and outcomes based on this prior learning.

## Conclusions

In conclusion, our study is the first to demonstrate that cadavers can be used to create dislocation models and that these models are effective in increasing students’ understanding and confidence with joint relocation. They offer a significant advantage over other methods of teaching such as online learning modules and instructional cards. This study has also shown that lower fidelity models such as the skeleton model can be used as a substitute. It is then up to each institution to decide which method to use and this may be dictated by financial constraints and cadaver availability. Simple blended learning training modules based on these models could also be developed for doctors working in areas exposed to joint dislocations such as family doctor in rural practice where delays to relocation and compromised management would otherwise be expected.

## Abbreviations

ANOVA: Analysis of variance
Cronbach Alpha: Alpha coefficient of internal consistency
MBBS: Bachelor of Medicine and Bachelor of Surgery
